# In Vitro Terbinafine Susceptibility of Dermatophytes and Mycological Cure Outcomes from a Multicenter Phase III Onychomycosis Trial

**DOI:** 10.3390/jof12100739

**Published:** 2026-10-02

**Authors:** Mahmoud Ghannoum, Erin Marie San Valentin, Amir Tavakkol

**Affiliations:** 1 Department of Dermatology, Case Western Reserve University and University Hospitals Cleveland Medical Center, Cleveland, OH 44106, USA; 2 Moberg Pharma AB (publ), 167 51 Bromma, Sweden

**Keywords:** onychomycosis, mycological cure, *Trichophyton rubrum*, antifungal resistance, minimum inhibitory concentration

## Abstract

Background: Dermatophyte infections, including onychomycosis, affect millions worldwide. Terbinafine, an allylamine antifungal, remains a foundational method of treatment. Objective: We aim to evaluate the in vitro antifungal susceptibility of terbinafine against dermatophyte isolates obtained from patients participating in a Phase III onychomycosis clinical trial. Methods: A total of 506 viable dermatophyte baseline isolates were tested using the Clinical and Laboratory Standard Institute (CLSI) M38-A3 broth microdilution method. The minimum inhibitory concentration (MIC) distributions in baseline isolates were compared between isolates from patients who were randomized and treated (group 1: terbinafine, *n* = 218 and vehicle, *n* = 108) and isolates from patients who screen-failed and therefore were not treated (group 2, *n* = 180). In addition, baseline MICs from group 1 were linked to their end-of-study mycological cure (negative KOH microscopy and negative fungal culture) to determine the relationship between MICs and mycological cure in study patients. Results: Terbinafine demonstrated potent antifungal activity with an overall MIC range of 0.001 to ≥0.5 µg/mL, MIC_50_ of 0.008 µg/mL, and MIC_90_ of 0.016 µg/mL. MIC distributions were comparable between group 1 and group 2 isolates (*p* = 0.98). Mycological cure was achieved across the full range of baseline MIC values. Only 16 isolates (3.2%) exhibited elevated MIC values (≥0.5 µg/mL). Of these, five were from terbinafine-treated patients, of whom three had evaluable end-of-study mycology results; two of these three patients achieved mycological cure, providing preliminary evidence that elevated in vitro MIC may not preclude clinical response to topical terbinafine. To our knowledge, this is the first reported analysis linking individual baseline MIC values to mycological cure for topical terbinafine and suggests that the high local nail concentrations achieved by the topical formulation may exceed the MIC threshold for isolates with reduced susceptibility.

## 1. Introduction

Onychomycosis is the most common nail disorder, affecting approximately 10% of the general population and up to 50% of individuals over 70 years of age [1,2]. Dermatophytes, particularly *Trichophyton rubrum* and *Trichophyton mentagrophytes*, are responsible for most onychomycosis cases in North America and Europe [3,4]. Certain patient populations are at increased risk, including the elderly and individuals with certain conditions such as diabetes and peripheral vascular disease [5]. Untreated infections in higher risk populations can also lead to more serious complications including cellulitis and osteomyelitis [6]. Beyond physical complications, onychomycosis has been found to also significantly impact quality of life, affecting physical, psychological and social functioning [7], where visible nail dystrophy can serve as a constant reminder of underlying health conditions.

Oral antifungal treatments have been traditionally prescribed to treat onychomycosis. Terbinafine and itraconazole have been U.S. Food and Drug Administration (FDA)-approved, and fluconazole is available as an off-label option in the U.S [1]. Terbinafine, an allylamine antifungal agent, inhibits squalene epoxidase, a key enzyme in fungal ergosterol biosynthesis [8]. This mechanism leads to accumulation of toxic squalene and depletion of ergosterol, resulting in fungal cell death [9]. Terbinafine has demonstrated potent in vitro activity against dermatophytes and remains a first-line treatment option for onychomycosis, owing to its high efficacy and low relapse rate [9].

However, large-scale susceptibility data from well-characterized clinical trial populations remain limited. Furthermore, the absence of established clinical breakpoints for terbinafine against dermatophytes complicates the interpretation of minimum inhibitory concentration (MIC) data and their correlation with treatment outcomes [10]. Systematic surveillance using standardized methodology in geographically diverse, prospectively enrolled patient populations is essential to establish baseline susceptibility patterns, monitor for emerging resistance, and inform treatment guidelines [11].

The MOB15B-IV trial, a Phase III, randomized, double-blind, vehicle-controlled study evaluating a novel topical terbinafine formulation for the treatment of distal subungual onychomycosis (DSO) across the United States and Canada, provided an opportunity to address this gap. The objective of this study was to determine the in vitro susceptibility of dermatophyte isolates obtained from patients who participated in this clinical trial against terbinafine for the treatment of onychomycosis, and to link baseline MIC values from the terbinafine-treated group to their end-of-study mycological cure outcome.

## 2. Materials and Methods

### 2.1. Isolate Collection and Identification

Clinical dermatophyte isolates were obtained from toenail samples collected from patients with clinically diagnosed and culture-confirmed onychomycosis participating in the MOB015B-IV Phase III study conducted at multiple sites across the United States and Canada (NCT05279846) [12]. Dermatophyte species were identified by phenotypic methods, which include macroscopic colony morphology and microscopic identification of reproductive structures. Molecular confirmation by ITS sequencing or MALDI-TOF MS was not performed. Accordingly, isolates that were phenotypically consistent with *T. mentagrophytes* were reported as “*T. mentagrophytes* complex” to acknowledge the taxonomic complexity within the group. Of the 365 patients enrolled in the study, 326 viable dermatophyte isolates (*n* = 218 isolates from terbinafine- and *n* = 108 isolates from vehicle-treated patients) and 180 isolates from non-randomized patients were available for susceptibility testing. A small number of patients contributed more than one isolate. For the mycological cure analysis, the unit of analysis was the patient outcome, as mycological cure was a patient-level outcome assessed at the end-of-study visit. For patients with more than one baseline isolate, the first available isolate was used.

### 2.2. Antifungal Susceptibility Testing

MIC testing was performed according to the Clinical and Laboratory Standards Institute (CLSI) M38-A3 reference method for broth dilution antifungal susceptibility testing of filamentous fungi [13]. Dermatophyte isolates were subcultured from frozen slants onto Difco™ Potato Dextrose Agar (PDA, BD Franklin Lakes, NJ, USA) and checked for purity. Inocula were prepared by harvesting conidia into sterile water and adjusting concentrations to 1–3 × 10^3^ conidia/mL using hemocytometer counts.

Terbinafine powder was dissolved in dimethyl sulfoxide (DMSO, Sigma-Aldrich, St. Louis, MO, USA) to prepare a stock solution, which was further diluted in Roswell Park Memorial Institute (RPMI) 1640 medium buffered with 3-(N-morpholino) propanesulfonic acid (MOPS) (United States Biological Life Sciences, Salem, MA, USA) to achieve final test concentrations ranging from 0.001 to 0.5 µg/mL. Microdilution plates were incubated at 35 °C for four days. The MIC endpoint was determined by visual inspection and defined as the lowest terbinafine concentration producing ≥80% growth inhibition compared to the drug-free growth control. Isolates with MIC values at the upper limit of the tested concentration range were reported as ≥0.5 µg/mL. Quality control was performed according to the CLSI M38-A3 guidelines.

### 2.3. Statistical Analysis

MIC distributions were compared between group 1 and group 2 using the Mann–Whitney U test. Isolates with MIC at the upper limit of the tested range were reported as ≥0.5 µg/mL (right-censored). The proportion of isolates with elevated MIC (≥0.5 µg/mL) was compared between groups using Fisher’s exact test. Species distributions across groups were compared using the chi-square test. All statistical computing and graphics analyses were performed using R (version 4.5.2) and RStudio (version 2026.01.0+392).

## 3. Results

A total of 567 dermatophyte isolates were identified from patients who were screened for participation in the MOB015B-IV study. Of these, 506 (89%) yielded viable growth upon subculture and were tested for terbinafine susceptibility prior to any treatment (baseline). Viable isolates were stratified into two groups: group 1 comprised isolates from patients who were subsequently randomized, i.e., received terbinafine treatment (*n* = 218 isolates) or vehicle treatment (*n* = 108 isolates) (total *n* = 326 isolates), while group 2 comprised isolates from patients who screen-failed and were not randomized (*n* = 180 isolates). The species distribution across all 506 viable isolates was *Trichophyton rubrum* (94%), *T. mentagrophytes* complex (4%), *E. floccosum* (2%), and *T. tonsurans* (<1%).

To evaluate whether restricting susceptibility testing to the randomized population versus patients who were not randomized would alter the susceptibility profile, the MIC distributions were first compared between group 1 and group 2. The two groups showed essentially identical distributions (MIC_50_ 0.008 µg/mL and MIC_90_ 0.016 µg/mL for both groups, *p* = 0.98). MIC_50_ is defined as the concentration that inhibited 50% of the isolates, while MIC_90_ refers to the concentration that inhibited 90% of the tested isolates. The proportion of isolates with an elevated MIC (≥0.5 µg/mL) was 2.5% (8/326) among the randomized patients (group 1) and 4.4% (8/180) among the screen-failure patients (group 2) (Supplementary Table S1), with an overall prevalence of 3.2% (16/506) across all tested isolates (*p* = 0.29). The remaining 96.8% exhibited MIC values below 0.5 µg/mL. Species distributions were also comparable between groups *(p =* 0.23). These findings confirm that the randomized and screen-failed populations (i.e., treated or untreated) in this study represented the same underlying dermatophyte distribution, supporting pooled analysis of the 506 baseline samples.

Terbinafine showed potent in vitro antifungal activity against all dermatophyte species tested (Table 1). The overall MIC range was 0.001 to ≥0.5 µg/mL, with MIC_50_ and MIC_90_ values of 0.008 and 0.016 µg/mL, respectively. *Epidermophyton floccosum* (*n* = 9) showed slightly higher MIC_50_ (0.016 µg/mL) and MIC_90_ (0.03 µg/mL) values than the *Trichophyton* species. Geometric mean MICs were similar across the species, ranging from 0.0079 µg/mL to 0.0122 µg/mL. These findings indicate that the terbinafine susceptibility is comparable across different species. On the other hand, the modal MIC was 0.008 µg/mL for *T. rubrum, T. mentagrophytes* complex, and *T. tonsurans.* Although the sample size is small, these values are consistent with prior reports of modestly reduced terbinafine susceptibility in this genus [14]. However, all *E. floccosum* MICs remained within the susceptible range (≤0.03 µg/mL), and the small sample size precluded precise percentile estimation.

**Figure 1:**
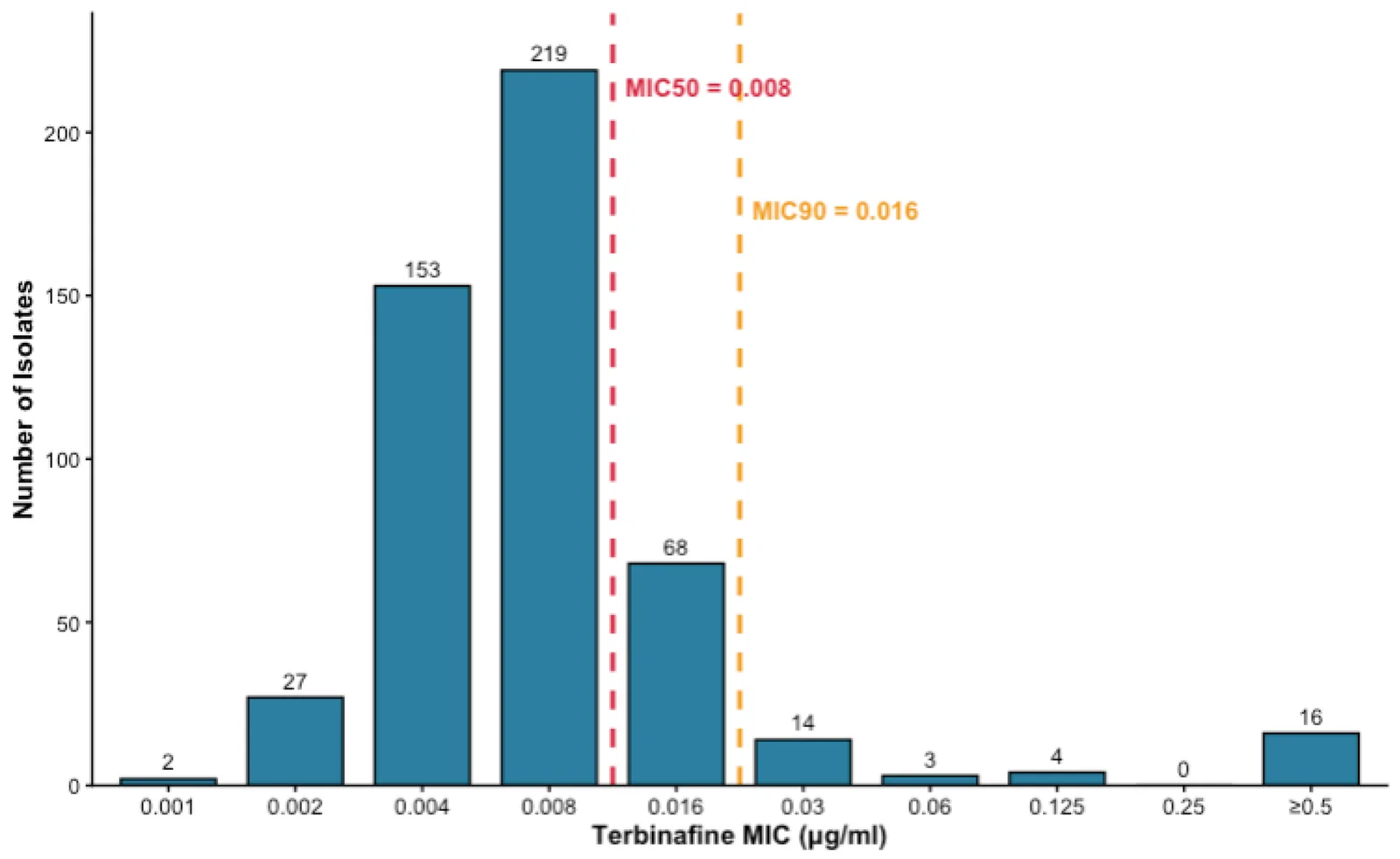
Distribution of terbinafine MICs across all dermatophyte isolates (*n* = 506). Dashed vertical lines mark the MIC_50_ (red, 0.008 µg/mL) and MIC_90_ (orange, 0.016 µg/mL), defined as the MIC values at or below which 50% and 90% of isolates, respectively, were inhibited. A total of 16 isolates (3.2%) had MIC values ≥ 0.5 µg/mL.

The MIC distribution across all dermatophyte isolates (*n* = 506) showed a unimodal distribution centered at lower MIC values, with about 75% of isolates inhibited at MIC ≤ 0.008 µg/mL and 90% inhibited at MIC ≤ 0.016 µg/mL (Figure 1). Elevated MIC values (≥0.5 µg/mL) were observed in only 16 isolates (3.2%), comprising 15 *T. rubrum* isolates and 1 *T. mentagrophytes* complex. Importantly, all isolates with elevated MICs were from baseline screening samples prior to any terbinafine or vehicle treatment, indicating pre-existing reduced susceptibility rather than treatment-induced MIC elevation in this small number of isolates.

**Figure 2:**
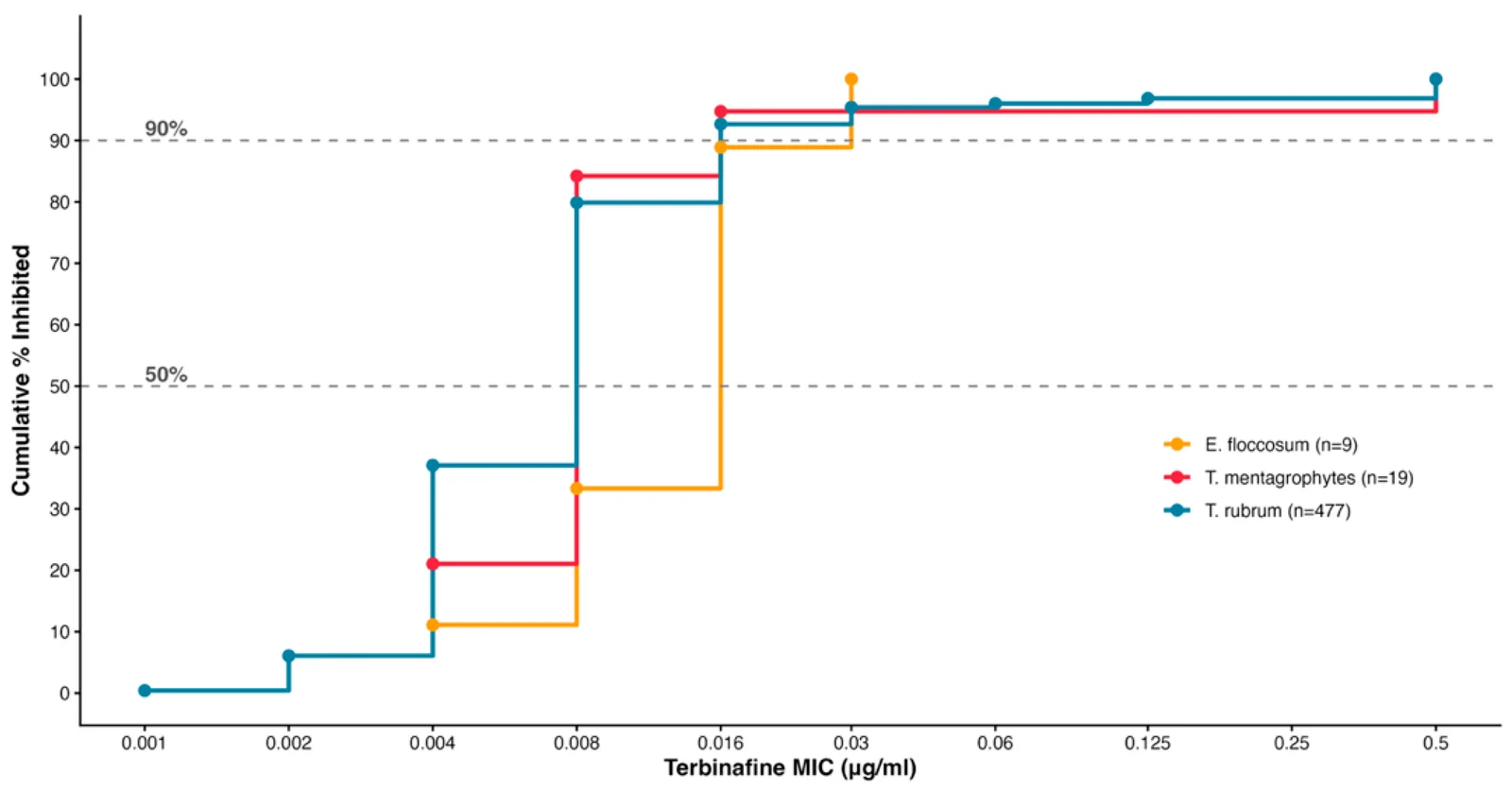
Cumulative percentage of isolates inhibited by terbinafine by fungal species (*n* = 505). All three species demonstrated similar susceptibility profiles, with 90% of isolates inhibited by ≤0.03 µg/mL. *T. rubrum* and *T. mentagrophytes* complex each contained isolates with MIC ≥ 0.5 µg/mL, while *E. floccosum* showed no elevated MIC values. A single *T. tonsurans* isolate with MIC 0.008 µg/mL is not plotted in this figure.

To evaluate whether terbinafine susceptibility differed by species, cumulative inhibition curves were generated for each species (Figure 2). The curves demonstrated similar profiles, with *T. rubrum*, *T. mentagrophytes* complex, and *E. floccosum* all crossing the 50% inhibition threshold at approximately 0.008 µg/mL. All three species reached 90% inhibition by 0.03 µg/mL. *E. floccosum* was the most uniformly susceptible, with none of its isolates exhibiting MIC ≥ 0.5 µg/mL.

**Figure 3:**
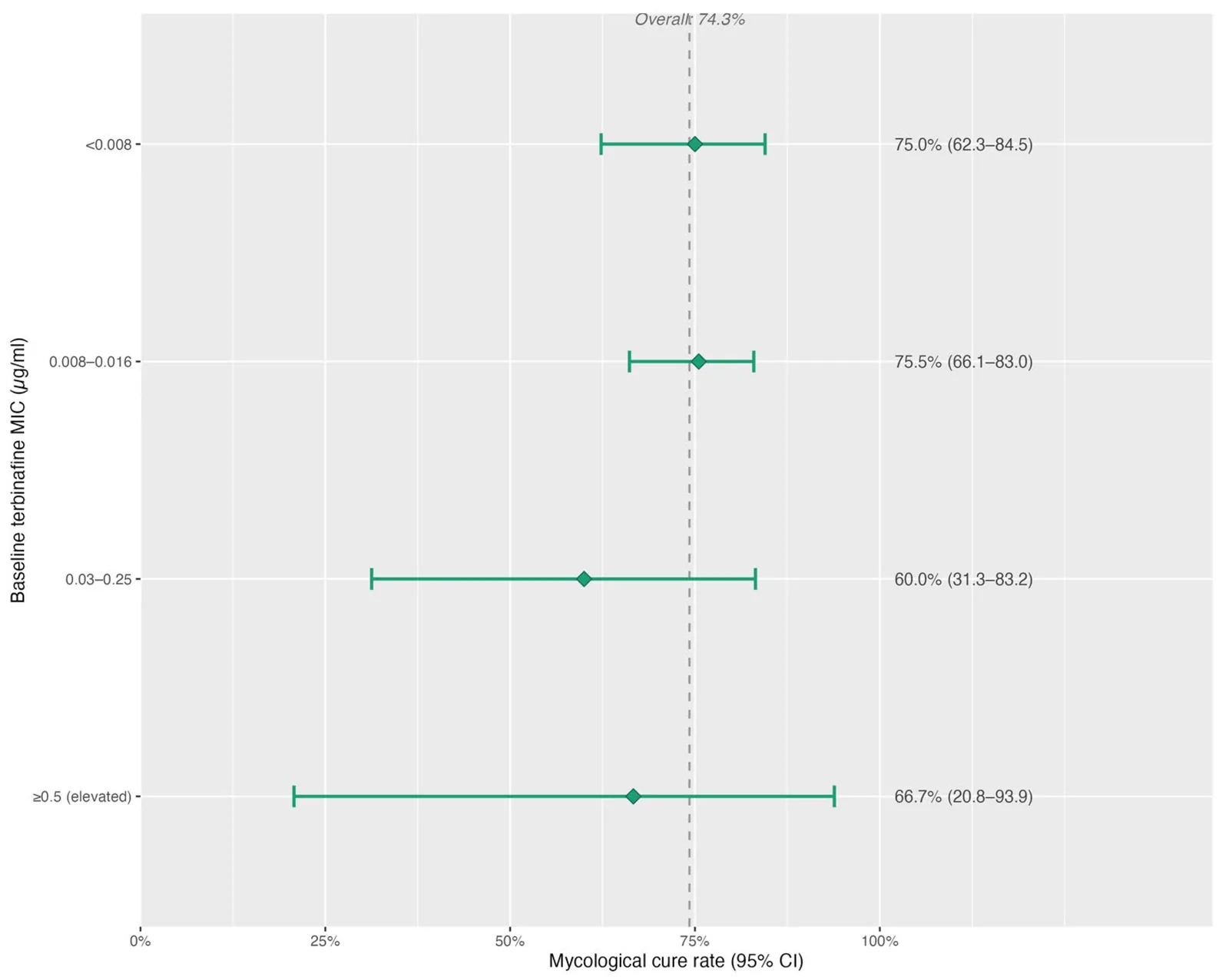
Mycological cure rate by baseline terbinafine MIC category. Analysis was restricted to terbinafine-treated patients with evaluable end-of-study mycology results (*n* = 167). Each diamond shows the cure rate point estimate for a given baseline MIC category, with horizontal bars representing the 95% confidence intervals (CIs). The dashed vertical line marks the overall mycological cure rate (74.3%) across all evaluable patients. Cure rates were similar across MIC categories including isolates with elevated baseline MICs (*≥* 0.5 µg/mL).

To assess whether the in vitro MIC values were predictive of clinical outcome, baseline MICs were linked to end-of-study (EOS) mycological cure, defined as both negative KOH microscopy and negative fungal culture. Because mycological cure is a patient-level outcome, this analysis used patients rather than isolates as the unit of analysis. This analysis included 167 of 215 terbinafine-treated patients with evaluable end-of-study results. The overall mycological cure rate was 74.3% (124/167, 95% CI 67.1–80.3%), consistent with the previously reported trial outcome [12]. Among 167 terbinafine-treated patients with evaluable mycology results, baseline MIC categories were distributed as follows: <0.008 µg/mL (*n* = 56), 0.008–0.016 µg/mL (*n* = 98), 0.03–0.25 µg/mL (*n* = 10), and ≥0.5 µg/mL (*n* = 3). Cure rates stratified by baseline MIC category were high across various MIC values, determined as follows: <0.008 µg/mL, 75.0% (42/56, 95% CI 62.3–84.5%); 0.008–0.016 µg/mL, 75.5% (74/98, 95% CI 66.1–83.0%); 0.03–0.25 µg/mL, 60.0% (6/10, 95% CI 31.3–83.2%); and ≥0.5 µg/mL (elevated), 66.7% (2/3, 95% CI 20.8–93.9%) (Figure 3). However, in those with an elevated baseline MIC of ≥0.5 µg/mL, the small number of isolates limits broad conclusion for this group but provides preliminary data.

## 4. Discussion

This study evaluated the in vitro susceptibility of 506 dermatophyte isolates to terbinafine using standardized CLSI methodology. Terbinafine demonstrated potent antifungal activity with MIC_50_ and MIC_90_ values of 0.008 and 0.016 µg/mL, respectively, consistent with previously published susceptibility data [15,16]. The MIC distribution in this study was comparable to that reported by Ghannoum et al. [17] for naftifine hydrochloride, another allylamine antifungal, against 350 dermatophyte strains. Both allylamines exhibit similar mechanisms of action through the inhibition of squalene epoxidase. Notably, ‘elevated MIC’ as defined in this study (≥0.5 µg/mL) reflects reduced in vitro susceptibility but should not be equated with clinically or genetically defined resistance. True terbinafine resistance requires molecular confirmation of SQLE mutations or validation against established breakpoints, neither of which were performed in this study.

In this study, only 3.2% of baseline isolates demonstrated MIC values ≥0.5 µg/mL, with similar proportions observed in the randomized (2.5%) and screen-failure (4.4%) subpopulations, respectively. Notably, these isolates were collected at the screening visit prior to any study treatment, demonstrating that elevated MIC values represent pre-existing reduced susceptibility rather than treatment-induced resistance in this small group. Moreover, the clinical significance of these elevated MIC values remains uncertain as epidemiological cutoff values (ECVs) and clinical breakpoints for terbinafine against dermatophytes have not been established. Figure 2 shows a species-specific profile of MIC values. *T. rubrum* and *E. floccosum* demonstrate a more uniform susceptibility, while *T. mentagrophytes* complex exhibits a wider MIC range. Specifically, the MIC_90_ for *T. mentagrophytes* complex remained at 0.016 µg/mL, although a single isolate in this species showed the highest observed MIC of ≥0.5 µg/mL. This single elevated MIC should not be interpreted as proof of a wider population-level susceptibility distribution in *T. mentagrophytes* complex, as larger sample sizes would be needed to characterize the true MIC distribution. These findings are also consistent with the prior literature demonstrating that *T. rubrum* strains are generally more susceptible to antifungal agents than *T. mentagrophytes* complex species [18]. Studies of *E. floccosum* have similarly documented low variation, with consistently low MICs for terbinafine [14]. The wider MIC range in *T. mentagrophytes* complex may reflect a broader susceptibility range within this species complex, encompassing multiple genotypes with varying resistance profiles [10,19]. Elevated MIC_90_ values for terbinafine (up to 8 mg/L) have been documented in a large-scale study (*n* = 498) of *T. mentagrophytes*-*interdigitale* complex isolates from India [20]. Notably, isolates from that study were primarily derived from cutaneous specimens rather than nails, which may limit direct comparison to onychomycosis-derived isolates. Nonetheless, various studies show that *T. rubrum* remains the primary cause in onychomycosis in Europe and North America, accounting for the majority of dermatophyte isolates in published studies [21,22,23].

A novel aspect of this study that has not been previously reported was our ability to link individual baseline MIC values to mycological cure outcomes following terbinafine treatment. Among 167 enrolled patients in the terbinafine arm with evaluable end-of-study mycology results, mycological cure rates were comparable across baseline MIC categories (Figure 3). Notably, two of three patients with elevated baseline MICs (≥0.5 µg/mL) achieved mycological cure despite reduced in vitro susceptibility. This high cure rate, despite elevated MICs, may be attributed to the high local terbinafine concentrations achieved with topical application, which is more likely to exceed the MIC threshold even for isolates with reduced susceptibility. While the small number of patients with an elevated MIC limits definitive conclusions, this observation suggests that standard broth microdilution MIC values may not directly predict treatment response for topical terbinafine. Furthermore, treatment failure in onychomycosis may be influenced by host-related factors independent of in vitro susceptibility, including nail growth rate, patient adherence, and disease severity [24]. It should also be noted that mycological cure rate data were available for only 167 of 215 enrolled terbinafine-treated patients. The remaining 48 (early terminations and patients with incomplete follow-up) could not be included in mycological cure analysis. The complete-case analysis, which included only patients who provided data for negative fungal culture of dermatophytes and negative direct KOH microscopy at Week 52, resulted in a 74.3% mycological cure rate that was similar to the previously published 69.9% (95% CI: [63.4, 75.9]) reported in the Phase III trial [12], which used the last-observation-carried-forward approach for replacement of missing values. Another limitation of this study is the use of baseline isolates only. Although approximately 30% of patients did not achieve mycological cure, most failures were due to positive microscopy with negative fungal cultures. Only 2 of 218 terbinafine-treated toenails had positive dermatophyte cultures at the end-of-study visit (Week 52); however, both isolates failed to produce viable growth in the susceptibility assay. Consequently, no post-treatment MIC data could be obtained, precluding assessment of treatment-emergent resistance or changes in MIC following terbinafine exposure. Our findings thus reflect pre-existing susceptibility patterns in this population.

It is known that terbinafine exerts its antifungal activity by inhibiting squalene epoxidase (SQLE), a key enzyme in the ergosterol biosynthesis pathway. Resistance to terbinafine has been primarily attributed to point mutations in the SQLE gene, with amino acid substitutions at positions Leu393 and Phe397 most frequently reported [25]. The Phe397Leu mutation has been associated with MIC elevations of 10- to 100-fold in clinical isolates [24]. In contrast to our findings, where only 1 of 19 *T. mentagrophytes* isolates (5.3%) exhibited MIC ≥ 0.5 µg/mL, studies from the Indian subcontinent have reported resistance rates exceeding 70% in *T. mentagrophytes* complex isolates, largely attributed to point mutations in the SQLE gene [26]. Most importantly, a subset of resistant isolates have exhibited elevated MICs in the absence of SQLE mutations which may suggest the presence of alternative resistance mechanisms [27]. In the present study, molecular characterization on the *Trichophyton* strains was not performed; therefore, we could not determine whether the 16 isolates with elevated MICs harbored SQLE mutations. Future studies incorporating whole-genome sequencing or targeted SQLE gene analysis are warranted to elucidate the genetic basis of reduced terbinafine susceptibility in North American populations and to monitor for the emergence of resistant species.

This study has some limitations. First, the species distribution was skewed toward *T. rubrum* (94%), with small numbers of *T. mentagrophytes* complex (*n* = 19), *E. floccosum* (*n* = 8), and *T. tonsurans* (*n* = 1) isolates, limiting conclusions about susceptibility patterns in non-*T. rubrum* species. However, such species distributions are typical of onychomycosis trials. Second, species identification was based on phenotypic methods without molecular confirmation, which is particularly relevant for the *T. mentagrophytes* complex where phenotypic methods cannot reliably distinguish *T. mentagrophytes*, *T. interdigitale*, and *T. indotineae*, which are species with potentially different susceptibility profiles. Third, molecular characterization of isolates with elevated MICs was not performed; therefore, we could not determine whether these isolates harbored SQLE mutations associated with terbinafine resistance. Fourth, only three patients with elevated baseline MICs had evaluable mycological cure data, precluding definitive conclusions about the clinical significance of elevated MIC values. Finally, only baseline (pre-treatment) isolates were tested; because post-treatment isolates were not available due to the high mycological cure rate, we could not assess treatment-emergent resistance or MIC changes following terbinafine exposure.

## 5. Conclusions

This study demonstrates that terbinafine shows potent in vitro activity against dermatophyte isolates from the U.S. and Canada cases of onychomycosis, with only 3.2% exhibiting elevated MIC values. Importantly, mycological cure was achieved by patients across the full range of baseline MIC values, including two of three patients with elevated baseline MICs of ≥0.5 µg/mL. This suggests that the reduced in vitro susceptibility did not necessarily translate into treatment failure with topical terbinafine. These data support the continued role of terbinafine as a first-line antifungal while also highlighting the importance of ongoing susceptibility surveillance given the global emergence of resistant *Trichophyton* species. These data establish a baseline susceptibility distribution in this North American trial population and provide preliminary evidence that elevated baseline MICs may not preclude mycological cure with topical terbinafine, although larger studies are needed to confirm this observation.

## Figures and Tables

**Table 1:** Minimum inhibitory concentration (MIC) values of terbinafine against dermatophytes. MIC_50_ and MIC_90_ show the concentrations that inhibited 50% and 90% of the strains tested, respectively. MIC_50_ and MIC_90_ were not calculated for *T. tonsurans* (*n* = 1) due to the insufficient sample size.

Species	*n*	MIC Range (µg/mL)	MIC_50_ (µg/mL)	MIC_90_ (µg/mL)	Geometric Mean	Mode
*T. rubrum*	477	0.001–≥0.5	0.008	0.016	0.0079	0.008
*T. mentagrophytes* complex	19	0.004–≥0.5	0.008	0.016	0.0092	0.008
*E. floccosum*	9	0.004–0.03	0.016	0.03	0.0122	0.016
*T. tonsurans*	1	0.008	-	-	-	-
All dermatophytes	506	0.001–≥0.5	0.008	0.016	0.008	0.008

## Data Availability

The original contributions presented in this study are included in the article/supplementary material. Further inquiries can be directed to the corresponding authors.

## Supplementary Materials

The following supporting information can be downloaded at: https://www.mdpi.com/article/10.3390/jof12100739/s1, Table S1: Distribution of terbinafine Minimum Inhibitory Concentrations (MICs) across study groups.
