# Supplementary material for: In Vitro Terbinafine Susceptibility of Dermatophytes and Mycological Cure Outcomes from a Multicenter Phase III Onychomycosis Trial
Source: J Fungi (Basel). 2026 Oct 2;12(10):739. doi: 10.3390/jof12100739 (PMC13706075; doi:10.3390/jof12100739)
Supplement: Supplementary file 1 [file jof-12-00739-s001.zip › jof-4540909-supplementary.pdf]

Supplementary Table S1. Distribution of terbinafine Minimum Inhibitory Concentrations (MICs) across study groups.

Group	Description	n	MIC Range	MIC_{50}	MIC_{90}	Geometric Mean	Elevated MIC, n (%)
**Group 1**	Randomized (Terbinafine+ Vehicle)	326	0.001–≥0.5	0.008	0.016	0.0078	8 (2.5%)
**Group 2**	Screen Failure	180	0.002–≥0.5	0.008	0.016	0.0083	8 (4.4%)
**Total**		506	0.001–≥0.5	0.008	0.016	0.008	16 (3.2%)
